# Diagnostic Accuracy of Abdominal Point of Care Ultrasound in Primary Care: Study Design and Protocol

**DOI:** 10.24908/pocus.v9i1.16987

**Published:** 2024-04-22

**Authors:** Antonio Calvo Cebrián, Rafael Alonso Roca, Ignacio Manuel Sánchez Barrancos

**Affiliations:** 1 Robledo de Chavela Primary Care Health-Center, Madrid Health Service, Madrid Spain; 2 Mar Báltico Primary Care Health-Center. Madrid Health Service Madrid Spain; 3 Pio XII Primary Care Health-Center, Castilla-La Mancha Health Service Ciudad Real Spain

**Keywords:** abdominal point of care ultrasound, family physician, diagnostic accuracy, primary care, medical history taking

## Abstract

The aim of this study is to estimate the diagnostic accuracy of abdominal point of care ultrasound (POCUS) performed by family physicians (FPs) in primary care (PC), in comparison with the findings in the medical record (MR) at 12 months of follow-up. This study is conducted entirely in PC healthcare centers in Spain. Abdominal ultrasound scans performed by FPs (selected on the basis of their ultrasound knowledge and experience) are compared with the findings, or not, in the patient's MR after a 12-month follow-up period. The study will involve 100 FPs in Spain and an estimated sample size of 1334 patients who are to undergo abdominal POCUS at the indication of their physician. The results of the abdominal POCUS will be collected and compared with the findings of the MR. This comparison will be performed by another physician of the research team, different from their FP after one year of follow-up. The diagnostic accuracy of abdominal POCUS has been addressed in the hospital setting but not in PC. This lack of evidence can begin to be resolved with studies such as the one we present, designed for unselected populations such as those treated in PC and taking the patient's MR as the gold standard, which will allow us to make comparisons with the patient's clinical course.

## Background

Abdominal point of care ultrasound (POCUS) has been employed as a diagnostic tool by family physicians (FPs) for years both in Spain and globally. Furthermore, literature has described curricula for training in this technique 1. Specifically, the Madrid Health Service has developed a training program of which some authors have contributed to teaching teams, providing instruction in the acquisition of abdominal POCUS skills. In other parts of Spain, there is an uneven advancement in the implementation of ultrasound equipment and training programs, although the scientific societies of family medicine (FM) in Spain provide access to POCUS training programs to FP professionals nationwide.

Several studies have demonstrated a strong correlation in POCUS interpretation between FP and hospital specialists other than radiologists, with concordances reaching up to 93% (95% CI 87-99%) 2, 3. Similarly, correlations between FPs and radiologists have been reported in studies made in Spain, with Kappa indexes above 0.8 4, 5.

In the assessment of diagnostic tests, it is crucial to calculate the likelihood ratio (LR). The LR is independent of disease prevalence and informs us about the probability that a patient has or does not have the disease given a positive or negative result. Evidence-based guidelines for physical examination recommend utilizing diagnostic or exploratory tests based on this information 6, 7.

Therefore, it is important to assess the diagnostic accuracy of POCUS in cases where FPs utilize it for a specific clinical suspicion and compare it to the findings of the patient's medical records (MRs) during medium-to-long term follow-up. However, there is a limited number of studies conducted in primary care (PC) settings 8.

In PC, it is crucial to optimize time. The use of POCUS during the clinical act of the medical practitioner takes additional time. Quantifying the effectiveness of ultrasound for diagnosing certain pathologies could support its practical use. Although there are studies in other fields on this subject, there are a lack of studies examining diagnostic accuracy in abdominal pain 9, 10, 11, 12, 13.

Performing abdominal ultrasounds on asymptomatic adult patients can result in discovering lesions in 22% of those explored. Merely 3% necessitate therapy after a two-year follow-up 14. In a similar study limited to elderly patients, 31% of ultrasound findings went undetected during conventional physical examinations 15. Another study conducted by German FPs designed for the early detection of renal cell carcinoma found that ultrasound examinations exclusively limited to the kidneys yielded a positive predictive value of 50% for positive findings and only 2% for equivocal findings, while identifying a rate of 12% of unexpected results 16. On the contrary, a study conducted on 1962 patients and covering ten common clinical scenarios found that 63% of cases did not require additional diagnostic techniques, and only 5% were wrongly classified as negative 17.

Generally, we speak of POCUS when we refer to the ultrasound performed by FPs on the patients they treat. In Spain, some FPs have been performing ultrasounds for over 20 years. Initially, ultrasounds were conducted following the systematic exploration carried out by radiologists. FPs still follow this approach when performing ultrasounds, which is also taught to some clinicians. We refer to it as a 'comprehensive exam'. POCUS has developed over time as an ultrasound performed by clinicians to answer clinical questions and rule in or rule out the presence of certain pathologies in specific scenarios. This type of ultrasound is commonly referred to as a 'focused exam'.

During a comprehensive abdominal ultrasound, it is possible to uncover lesions in organs that do not exhibit symptoms, resulting in a diagnostic cascade of multiple tests. Despite this, the added concern for the patient remains a top priority. It should be noted, however, that clinical consequences of abnormal ultrasound findings occur rarely 18, 19, 20. These may result in the use of other imaging methods that emit ionizing radiation or invasive procedures that offer no benefit to the patient's health and may have significant adverse effects.

Quantifying overdiagnosis may prompt a reassessment of ultrasound performance by clinicians. This can lead to more symptom-focused examinations like POCUS or more studies.

In fact, one systematic review on ultrasound in PC has determined that symptom-focused ultrasound scans at the point of care are linked to increased diagnostic accuracy, reduced overdiagnosis and underdiagnosis rates, and lower technical skill training requisites. However, the review also indicates that these results are area-specific, and further research is necessary to substantiate the usefulness of diagnostic accuracy within the scope of FPs' duties. It highlights the insufficient quality of the research conducted and underscores the necessity for further studies. Additionally, it is important to determine the adequacy and quality of the training received by the FP, as well as to track the clinical progress of patients who undergo ultrasound by the FP 21.

Another review confirms the safety of POCUS use by FPs and indicates its positive impact on patient diagnosis. Nevertheless, the review highlights differences among populations and significant variability across clinical scenarios, suggesting that diagnostic accuracy must be assessed for each clinical entity and across different settings. There is a recommended need for PC studies, as the majority of ultrasound studies conducted by FPs originate from emergency settings 22.

This research project aims to provide evidence for all these questions.

The main objective of this study is to assess the diagnostic accuracy and validity of abdominal ultrasound performed by a FP in a PC environment. The patient's MR findings at one-year follow-up will be used as a reference test. Additionally, the study aims to determine the frequency with which FPs perform abdominal ultrasounds in different clinical scenarios, as well as the time spent in performing the technique and the identified ultrasound diagnosis. The diagnostic accuracy in each clinical scenario will be estimated, according to the type of ultrasound exam performed (focused or comprehensive), the professional's training, and the characteristics of the patient and the environment (rural or urban). We will examine the correlation between a FP’s diagnostic precision and several contributing factors, including their ultrasound scanner type, training experience, and past ultrasound experience. Additionally, we will estimate the incidence of overdiagnosis and underdiagnosis.

## Methods

This is a prospective observational study of diagnostic test accuracy. The recommendations of the STARD statement (https://www.equator-network.org/reporting-guidelines/stard/) were adhered to. The study will take place at PC health centers located in different autonomous communities of Spain over a period of two years, with 12 months for patient recruitment and 12 months for MR review. The research includes patients over 18-years of age visited by their FP and who undergo an abdominal POCUS. All participants are required to sign an informed consent document prior to undergoing an ultrasound administered by their FP at the health center; abide by the study protocol; and approve the consultation of their MR in a year. Recruitment of participants excludes pregnant women and those who have received an abdominal ultrasound from a radiologist in the past three months.

All FPs who participated in any training activities (including courses and scientific conferences developed by the Ultrasound Working Group of the Spanish Society of Family and Community Medicine) within the last ten years were invited to participate. They had to fulfill the following requirements: work at a PC center equipped with an ultrasound device, received at least 50 hours of theoretical and practical training in abdominal ultrasound, possess one year's experience in ultrasound, and had conducted at least 100 abdominal ultrasound examinations previously. The team of principal investigators thoroughly reviewed all prerequisites.

The study's sample size was calculated based on diagnostic tests studies with a sensitivity of 75% and a specificity of 90%. The researchers anticipated a prevalence of pathological findings of approximately 15%^23^, ^24^ and established a confidence level of 95% with a precision of 6%. The resulting size was 1334 participants, determined through the Epidat v 4.2 program. 100 sonographer FPs are included, each committed to recruiting between 20 and 50 patients for the study. The number of collaborators has been overestimated by 20%, resulting in a commensurate loss of collaborating physicians.

Demographic, ultrasound and technique variables, clinical variables, baseline test results, and variables associated with the investigating physician are all gathered and listed in Table 1.

**Table 1:** Study protocol variables.

**Sociodemographic Variables **	
· Age and gender	
**Ultrasound Device and Ultrasound Technique **	
· Type of ultrasound machine, Time in minutes taken to perform POCUS, Type of POCUS exam (Focused or Comprehensive exam).	
**Clinical Variables **	
· Clinical picture presented by the patient for abdominal ultrasonography (closed list of clinical pictures): Right upper quadrant abdominal pain, Dyspepsia, Hematuria, Palpable abdominal mass, Nephritic colic, Constitutional syndrome, Abnormal liver enzymes, Other analytical alterations suggesting abdominal pathology, Screening for Abdominal Aortic Aneurysm, Abdominal pain in another location, Abdominal trauma, Pelvic pain, Others.	
· Abdominal ultrasound result (negative-normal vs positive-pathological findings)	
· If positive, description of pathologic ultrasound findings (closed list): Abdominal Aortic Aneurysm, Aortic dissection, Aortic rupture, Dilated inferior vena cava, Pancreatic mass, Cholelithiasis, Cholecystitis, Common Bile Duct dilatation, Hepatomegaly, Choledocholithiasis, Vesicular polyposis, Enlarged gallbladder, Splenomegaly, Fatty liver, Ascites, Abdominal organ rupture, Hepatic space occupying lesion, Renal space occupying lesion, Renal cyst, Nephrolithiasis, Distal ureter dilatation, Hydronephrosis, Ureteral and bladder lithiasis, Bladder neoplasia, Prostatic volume, Distended bladder-acute urinary retention, Ureterocele, Bladder diverticulum, Struggling bladder, Urachal cyst, Acute appendicitis, Small bowel obstruction, Ectopic pregnancy, Uterine fibroids, Ovarian cyst, Ovarian mass, Others.	
Scale of action with respect to main pathologic finding	1. Requires referral to the emergency department and/or suspicion of malignancy 2. Preferential referral required 3. Requires normal referral or a complementary test 4. Requires treatment in primary care 5. Requires follow-up in primary care
Overdiagnosis (Yes or no)	Presence of ultrasound findings unrelated to the current diagnostic process and requiring further exploration or intervention.
**Outcomes**	
Pathologic findings in the medical record at 12-month follow-up:	At the end of the 12-month follow-up period, the medical history will be considered positive when it shows the presence of pathological diagnostic findings (that required intervention, i.e. other diagnostic techniques or medical or surgical treatment or follow-up), which are related to the initial process and are susceptible to diagnosis by ultrasound. The absence of such findings will be considered negative.
Underdiagnosis	Medical record reveals a clinical condition or diagnosis, detectable by ultrasound, that was not diagnosed in the initial ultrasound performed by their family physician and that justifies the initial picture.
**Family Physician**	
Number of accredited hours of training in abdominal POCUS, Number of POCUS examinations previously performed, Number of years of experience performing POCUS, Rural or urban environment, Healthcare area and Spanish autonomous region	

Baseline variables will be obtained through patient interviews and ultrasound examinations administered by the FP. Data will be collected in both a physical data collection notebook and a web-based form, and assigned an anonymous identifier. Ultrasound images will be stored within the machine, and the results will be recorded in the patient's MR and the data collection form (DCF).

**Figure 1:**
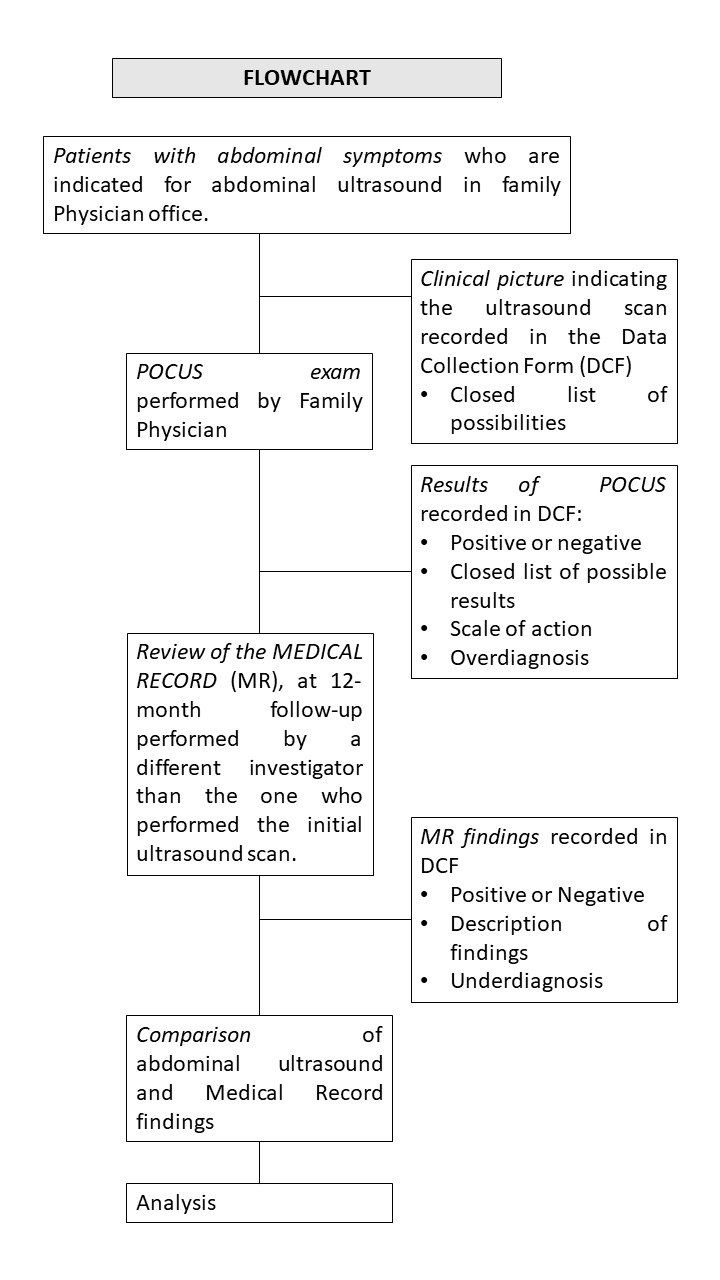
Flowchart depicting study protocol.

MRs will be reviewed 12 months after abdominal POCUS is conducted. The MR will be examined by an investigator from the research team, who is not the patient's regular physician, at the 12-month mark. Diagnostic findings consistent or inconsistent with the initial ultrasound diagnosis will be documented in both the patients' PC and hospital MR. The baseline variable "Findings in the MR at 12-month follow-up" will be recorded in the DCF. The study's actions are depicted in the flowchart (Figure 1).

### Diagnostic Procedure

**Figure 2:**
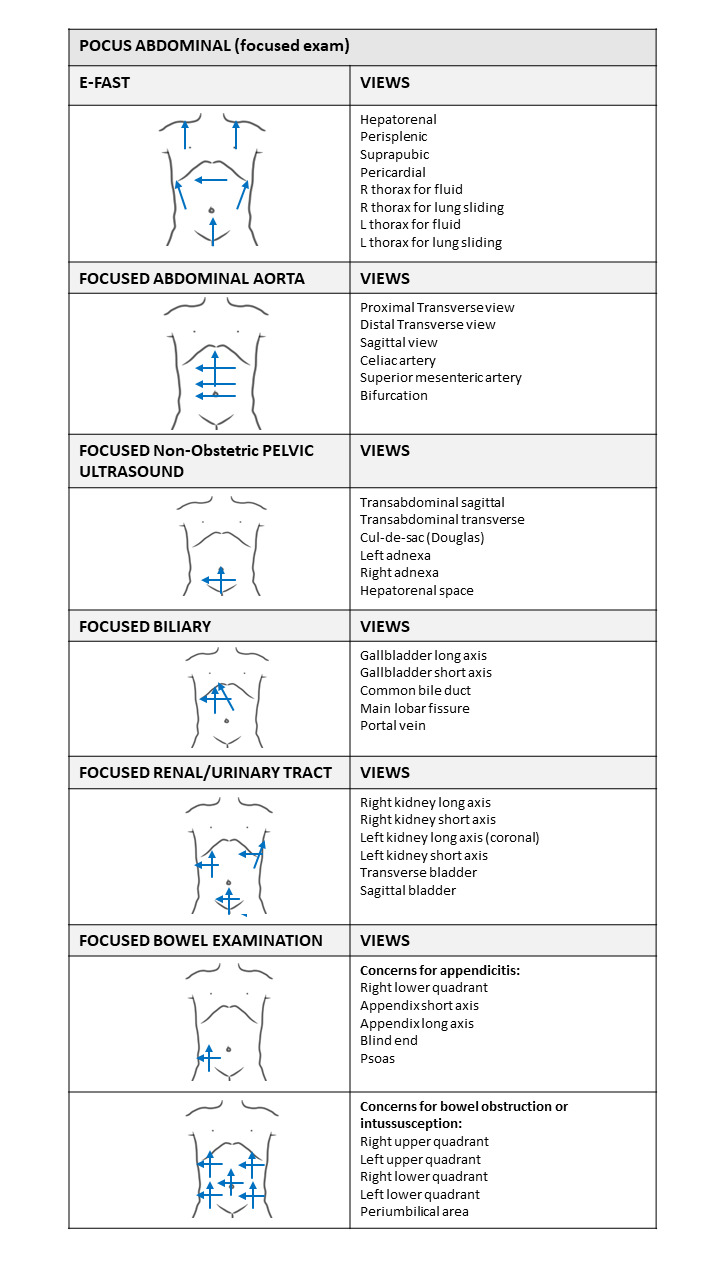
Abdominal-focused POCUS procedure recommended by theAmerican Academy of Family Physicians and the American College of Emergency Physicians 25, 26.

Patients presenting with abdominal pathology at their FP’s office and receiving an abdominal POCUS are offered the opportunity to participate in this study. All patients will receive information regarding the study's purpose and must provide informed consent per the study protocol. POCUS focused exams that are performed will adhere to the recommendations provided by the American Academy of Family Physicians and the American College of Emergency Physicians^25^, ^26^ as depicted in Figure 2. 

**Figure 3:**
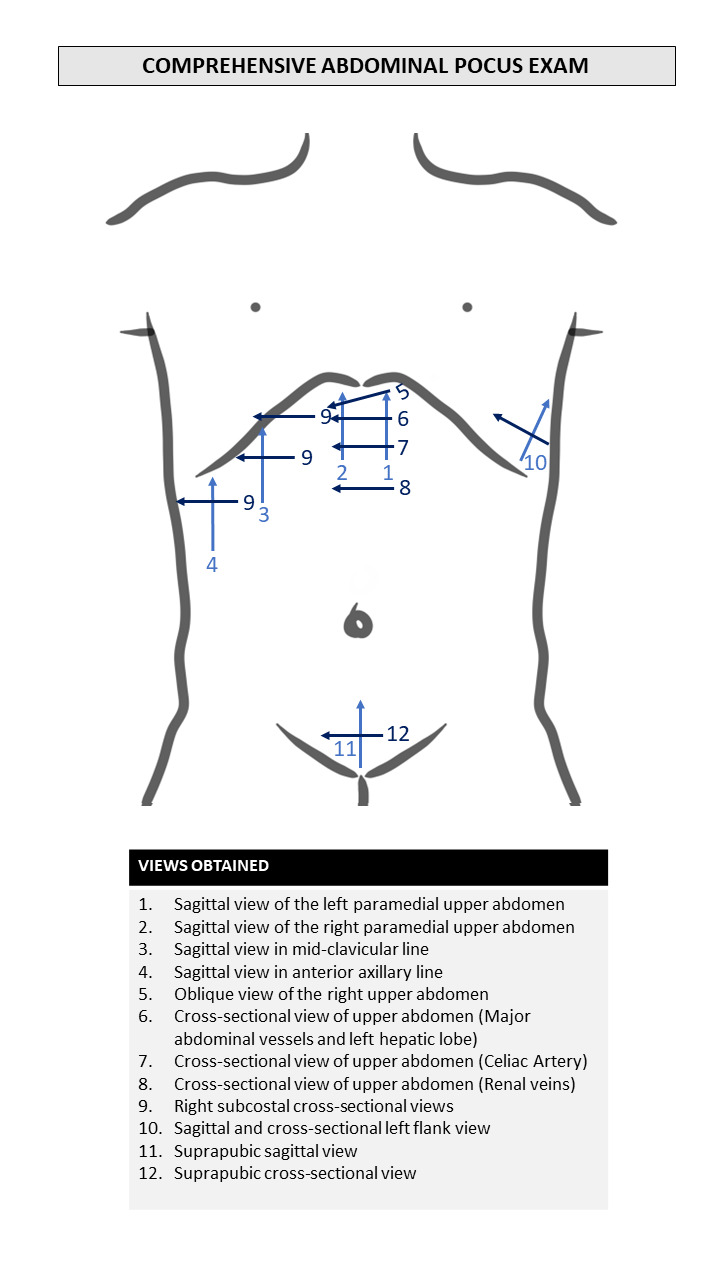
Comprehensive abdominal-focused POCUS procedure recommended byProfessor Segura Cabral 27.

For comprehensive POCUS, a complete examination of the abdomen will be conducted utilizing the traditional system of ultrasound views outlined by Professor Segura Cabral^27^ (shown in Figure 3). The performance of the test yields a result that will be labeled normal or abnormal. The diagnostic impression will then be recorded in the DCF.

### Statistical Analysis

The study will provide a description and estimation of patients' characteristics, physician involvement, clinical processes, and findings. Qualitative variables will be presented as percentages with a 95% confidence interval (95% CI), while quantitative variables will be presented as a mean and standard deviation or median and IQR (interquartile range) if they do not follow a normal distribution. The characteristics of patients, ultrasound techniques and types, and physicians will be compared based on normal or pathological diagnosis. Continuous variables will be analyzed using the student’s t-test for independent samples, while categorical variables will be analyzed using the chi-square test. Sensitivity, specificity, positive predictive value, negative predictive value, positive LR, and negative LR will be calculated, along with their corresponding 95% CIs. These calculations will be performed for all clinical scenarios, using FP ultrasound as index and the final diagnosis in medical history (MH) after one year of follow-up, as reference test. Additionally, the frequency of overdiagnosis and underdiagnosis will also be calculated, along with their corresponding 95% CIs. A multivariable logistic regression analysis will be utilized to identify the factors related to higher diagnostic accuracy.

## Discussion

This study is, to the best of our knowledge, the only diagnostic accuracy study performed in PC that will prospectively use the patient's clinical course (including eventual diagnoses) as a reference test to evaluate abdominal POCUS. Other studies in our setting were also descriptive and identified scenarios where the test was frequently used; however, they did not assess its accuracy in a PC setting 28. Furthermore, multiple concordance studies have been conducted with radiologists, but none have utilized as many investigators, or a large sample size as estimated in this study 2, 3, 4, 5, 29.

Although the diagnostic accuracy of various abdominal clinical scenarios has been studied in hospital settings, studies on this issue have not yet been conducted in PC. Thus, it is important to address the lack of evidence to support the proper use of this technique and to determine the optimal conditions for training, technology, and time use. Additionally, it is necessary to investigate whether the technique is being used in an optimal manner. 

### Strengths

It is important to note that the study will be conducted in the real-world setting of routine clinical practice, rather than simulated scenarios. The study will adhere to pragmatic principles and will maintain objectivity in its assessment. The purpose will be to evaluate the diagnostic abilities of PC physicians using POCUS, after receiving training from both Scientific Societies of Primary Care and Health Services of Autonomous Communities. This is a multicenter study with broad geographic representation, facilitating enhanced patient and professional participation from varied educational, occupational, and contextual backgrounds. The Ultrasound Working Group of the Spanish Society of Family and Community Medicine is leading this research and has vast experience in PC ultrasound education and practice. This is a reliable team that has done extensive work in training numerous FPs in ultrasound and organizing ultrasound conferences and congresses. Additionally, they have participated in various research projects and ultrasound publications in the field of FM.

### Limitations

Technology can introduce bias in the information. There are variations in ultrasound devices among different healthcare facilities, with each using unique devices acquired at specific times, leading to different performance levels. Diagnostic results may be influenced by different machines, so the data will indicate the type of ultrasound machine used. To ensure a blinded review of the reference test, MRs will be collected and evaluated by a different research team member, to minimize information biases. In order to minimize selection bias, it is important to consider the patients selected, as one researcher may have included significantly more cases than another. This could potentially skew the results towards those patients. To achieve balance in the number of patients per physician, each collaborator must obtain a minimum of 20 and a maximum of 50 cases. Additionally, every physician should extend an invitation to participate in the study to all patients who undergo an abdominal POCUS in their office. This will prevent the selection of patients with more severe pathologies or those that are easily diagnosed by ultrasound.

A follow-up period of 12 months has been established, which may overestimate the false-negative rate as new diagnoses may appear during this period that were not visible in the initial ultrasound. A 12-month follow-up period was established due to delays in accessing diagnostic tests and specialist visits within our healthcare system. Spain's healthcare system is publicly funded and universal, but due to high demand, waiting lists and delays for diagnostic tests and specialist consultations are common. This was considered during the study design, and a sufficient follow-up period was established to allow for the necessary diagnostic studies to be performed. The physician's clinical attitude will not be affected by the patient's participation in the study. The patient will undergo all necessary tests and referrals based on their clinical needs. To avoid time bias in acute pathologies related to the initial process, the MR will be reviewed 12 months after the ultrasound scan, taking into account any findings that occurred at one and three months. The variable MR has been defined precisely to only include positive MR findings that are related to the initial process and can be diagnosed by POCUS.

However, the development of the technique, access to training, and the availability of ultrasound devices varied among the regional healthcare services. The results will be categorized by autonomous communities and health areas.

### Potential Factors that May Predict Diagnostic Accuracy

To ensure homogeneity in the sample of collaborating FPs, those participating in the study must receive minimum accreditation for ultrasound training. We will gather data on the hours of abdominal ultrasound training to see if it impacts diagnostic accuracy. Additionally, we will collect information on the physician's prior experience, the number of exams performed before the study, and years of experience performing abdominal ultrasound. We understand that all these factors do influence a physician's ultrasound skill.

To minimize potential bias, we will provide objective definitions of MH outcomes.

In reviewing the MH at the 12-month mark, false negatives can result from an information bias when there is no diagnosis of diseases with a prolonged chronic course, episodic course that does not reoccur during that year, or diagnostic delays exceeding one year (waiting lists, rare pathologies, etc.). It could be debated that ultrasound performed by a radiologist instead of a healthcare provider after one year should be the gold standard. However, we think that this would imply going out of the usual clinical practice. In many of these situations, specifically when performing a focused ultrasound scan (POCUS), a comprehensive ultrasound is not requested. Nevertheless, we believe that any significant pathology would have become apparent after one year of follow-up, indicating that there was not an underdiagnosis.

## Conclusion

The study aims to provide objective evidence on the diagnostic accuracy of abdominal ultrasound performed by family physicians in their typical work settings. Currently, the evidence base for ultrasound in Family Medicine is scarce, necessitating further research to determine its usefulness and assess its varied applications. It is essential to ascertain the suitability of the training, technological, time, and geographical conditions, and their correlation with the diagnostic potential of the method.

## Ethical Approval

The study has been approved by the Clinical Research Ethics Committee of Puerta de Hierro Majadahonda University Hospital (reference number 96.23) and the Primary Care Research Commission of Madrid Region (reference number 20230011). All patients will give written informed consent for use of their personal and clinical data for research purposes.
